# Rheumatoid Arthritis-Associated Interstitial Lung Disease: Diagnostic Dilemma

**DOI:** 10.1155/2011/872120

**Published:** 2011-06-04

**Authors:** Mark J. Hamblin, Maureen R. Horton

**Affiliations:** Division of Pulmonary and Critical Care Medicine, Johns Hopkins University School of Medicine, 1830 East Monument Street, 5th Floor, Baltimore, MD 21205, USA

## Abstract

Interstitial lung disease (ILD) is an increasingly recognized complication of rheumatoid arthritis (RA) contributing to significantly increased morbidity and mortality. Diagnosis can be challenging since patients are unlikely to report dyspnea due to an overall decrease in physical activity with advanced arthritic symptoms. Additionally, infections, drug toxicity, and environmental toxins can mimic ILD, creating significant diagnostic dilemmas for the clinician. In this paper we will explore an effective clinical algorithm for the diagnosis of RA-ILD. We will also discuss features of drug-related toxicities, infections, and environmental toxins that comprise the main entities in the differential diagnosis of RA-ILD. Finally, we will explore the known and experimental treatment options that may have some benefit in the treatment of RA-ILD.

## 1. Introduction

It has been estimated that nearly 50% of rheumatoid arthritis (RA) patients will develop some form of respiratory abnormality during their lifetime [1, 2]. In fact, one study by Toyoshima et al. placed pulmonary disease as the second leading cause of death in RA accounting for nearly 20% of the mortality [3]. While there are a myriad of pulmonary complications associated with RA (Table 1), the most debilitating remains rheumatoid arthritis-associated interstitial lung disease (RA-ILD). Advancements in imaging technology have improved our ability to diagnose RA-ILD, and what once was thought to be a relatively rare complication is now suspected to affect 20–30% of RA patients [4]. 

In this paper, we will discuss the initial evaluation of suspected RA-ILD focusing on screening algorithms and radiologic features of the disease. However, screening and diagnosis of RA-ILD is only one aspect of care. Once the presence of RA-ILD is certain, the challenge for clinicians comes in distinguishing an exacerbation of RA-ILD from infection, drug toxicity, or a host of other comorbid disease entities. We will explore a practical approach to the workup and treatment of a clinical deterioration in the established RA-ILD patient, and the following case history will serve as a framework for this discussion highlighting some of the inherent challenges. 

## 2. Case Discussion

**Figure 1:**
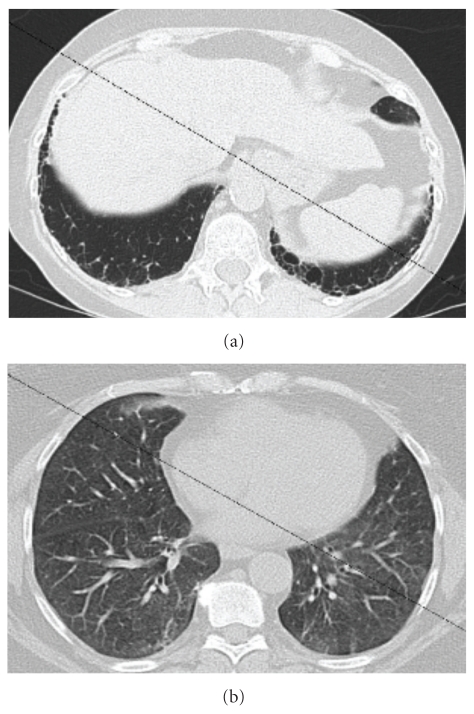
HRCT demonstrating predominantly reticular findings (a) as well as ground glass changes (b).

A 64-year-old woman was originally diagnosed with RA in 1986, and over the next several years her treatment regimen escalated from nonsteroidal anti-inflammatory medications with gold salts to D-penicillamine and methotrexate (MTX). In 2000, she was started on etanercept. Her arthritis symptoms were manageable on a regimen of etanercept and nonsteroidal anti-inflammatory medications, but after a few years of therapy she began to develop a mild non-productive cough but no noticeable dyspnea on exertion. In 2007, she was admitted to the hospital with acute renal failure. A kidney biopsy showed focal and segmental sclerosis. A chest X-ray from that hospitalization was abnormal, and a high-resolution chest CT (HRCT) demonstrated increased reticular markings consistent with an interstitial lung disease (Figure 1). She was started on peritoneal dialysis and prednisone for the renal failure but did not undergo any further evaluation of her pulmonary infiltrates. 

Her cough persisted, and she eventually became dyspneic climbing a flight of stairs. She was finally referred for an evaluation by a pulmonologist. Additional findings obtained at her initial visit included a history of myocardial infarction in 2001 and hypothyroidism treated with levothyroxine. She also kept two cockatiels as pets. She had no other hobbies or environmental exposures, and she was never a smoker. In addition to her cough, dyspnea, and arthritis pain, she also admitted night sweats over the past 6 months, but no weight loss. Her exam revealed a low-grade fever at 99.6 F, and she had bibasilar crackles but no digital clubbing. Her oxygen saturation was 100% at rest. Pulmonary function tests (PFTs) were obtained which demonstrated a preserved total lung capacity and forced vital capacity at 106% and 90% of predicted, respectively, but her diffusing capacity (DLCO) was moderately reduced at 61% of predicted. 

In summary, this was a woman with long-standing RA greatly increasing her risk for RA-ILD. However, she also had a history of using several medications (gold salts, D-penicillamine, methotrexate and etanercept) all of which have been associated with drug-induced interstitial pneumonitis. In addition, she had exposure to cockatiels that could induce an unrelated hypersensitivity pneumonitis. Her low-grade fevers and night sweats in the setting of chronic immunosuppression could also suggest a possible chronic infectious etiology or even an occult malignancy. Finally, she had a history of a myocardial infarction and renal failure, and she was now on peritoneal dialysis. She had no record of an echocardiogram since 2001, so a possible coexistent medical problem could account for her symptoms of dyspnea and cough. 

## 3. Establishing a Diagnosis of RA-ILD

Although our index case is representative of a standard referral to our practice, the majority of cases of RA-ILD are uncovered following patient complaints to their primary care physician or rheumatologist regarding the onset of progressive dyspnea on exertion. A growing number of rheumatology practices are facilitating a standardized screening algorithm for RA-ILD; however, the most cost-effective approach to screening has not been universally established [5]. 

In the era prior to the routine use of HRCT, the prevalence of RA-ILD was about 1–5% based on clinical exam and chest X-ray [6, 7]. In contrast, histological analysis of lung tissue from RA patients found the presence of pulmonary interstitial abnormalities in roughly 80% of patients [8]. A more recent clinical study employing HRCT also detected abnormalities in 80% of patients evaluated for dyspnea; however, only about 25% of these changes were consistent with ILD [9]. Additional findings included bronchiectasis, bronchiolitis, and pulmonary nodules. Similarly, other studies utilizing HRCT also found that only about 20–30% of symptomatic patients had ground glass opacities or increased reticular markings consistent with ILD [4, 10]. Given the poor sensitivity of chest X-ray and the high prevalence of abnormalities that do not always correlate to clinical disease on HRCT, we do not recommend imaging as an initial screening tool in asymptomatic patients.

Pulmonary function testing, and more specifically the DLCO, appears to be the most sensitive test available to screen for the presence of RA-ILD. Similar to our index case, Dawson et al. found that 80% of patients with RA had a reduced DLCO, while only 5–15% of patients had a purely restrictive defect on spirometry [10]. While the DLCO may be highly sensitive for RA-ILD, the specificity is lacking due to the prevalence of emphysema that can destroy vascular beds thus affecting the DLCO. In fact, Geddes et al. reported obstructive spirometry findings in more than 80% of RA patients in the form of bronchiectasis, constrictive bronchiolitis, or emphysematous changes [11]. These obstructive disorders also appear to be at least partially the effect of the disease process since obstructive airways disease in a nonsmoking RA population was much higher than a nonsmoking control population [12]. Additionally about 25% of patients have cricoarytenoid joint involvement, and although it is usually asymptomatic, it can evolve, sclerosing the joint leading to hoarseness, pain, and upper airway obstructive respiratory symptoms [13]. 

Despite the fact that emphysema alone may decrease the DLCO in the absence of RA-ILD, we still recommend follow-up imaging when the DLCO is reduced to less than 70% of predicted, especially in the absence of other abnormalities in the PFTs. This is based on the increasing recognition of “pseudo-normal PFTs” created by combined pulmonary fibrosis and emphysema in the idiopathic pulmonary fibrosis (IPF) population [14]. In several studies, up to 40% of patients had radiographic evidence of emphysema and pulmonary fibrosis on HRCT. However, they had FVC and TLC values that were normal or only had a borderline restrictive defect, and the FEV1/FVC ratio was often normal or only mildly reduced even with radiographic evidence of severe emphysema [14–16]. In this subset of patients, there was a uniform decrease in the DLCO (less than 40% in most studies) that was often out of proportion to the rest of the PFT abnormalities. Considering that there is a high prevalence of tobacco use in the RA population, this pattern of combined pulmonary fibrosis and emphysema may be just as common as that seen in IPF; however, further studies are needed [17].

Unfortunately, routine pulmonary function tests on every RA patient have not been established as a cost-effective approach to screening for pulmonary disease [18]. Considering that a number of patients will have abnormalities that do not correlate with clinical disease, we believe that the history and physical remain the vanguards of clinical evaluation until further studies of costeffectiveness are presented [11]. We advocate that each clinic visit include a brief inquiry into the presence of cough or mild dyspnea when climbing a flight of stairs or on an incline, as well as clinical examination for pulmonary crackles or digital clubbing although these are usually only found in advanced disease [19]. Additional patient factors that should increase the suspicion for RA-ILD include sex, anti-CCP levels, and smoking status. In epidemiologic studies, men are much more likely than women to develop RA-ILD [20]. High levels of anti-CCP antibodies have been associated with pulmonary fibrosis, and tobacco abuse (>25 pack years) may have the strongest association with the development of RA-ILD with an odds ratio of 3.8 [21, 22]. 

**Figure 2:**
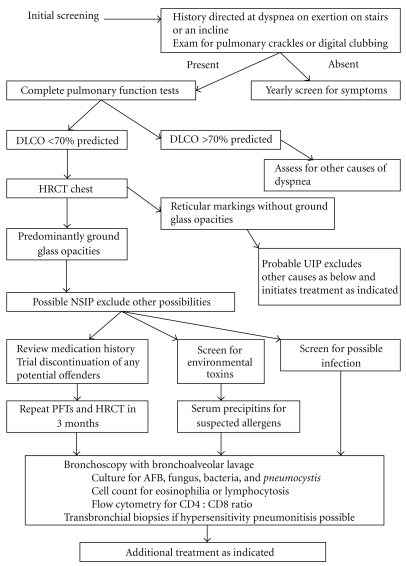
Diagnostic algorithm for the evaluation of rheumatoid arthritis.

In summary, if dyspnea or cough is present, a complete set of pulmonary tests is indicated including spirometry, lung volumes, and DLCO (Figure 2). A DLCO reduced to less than 70% of predicted should be the threshold for further imaging with HRCT.

## 4. Radiologic Evaluation and Histopathology

The presence of bibasilar, symmetrical, ground glass infiltrates, increased reticular markings, or honeycombing on HRCT is highly suggestive of ILD [23]. There can be a host of other findings including pleural involvement, rheumatoid nodules, bronchiectasis, emphysema, or even bronchiolitis with or without consolidation [24, 25]. In regards to RA-ILD, the radiologic findings do appear to correlate reasonably well with the histopathological subtype, and therefore they carry some prognostic significance.

Usual interstitial pneumonitis (UIP) is the predominant histological finding in RA-ILD affecting more than 50% of patients [26, 27]. UIP is the same histological subtype that underlies the end-stage lung disease idiopathic pulmonary fibrosis (IPF), and consequently it carries with it a relatively similar prognosis. It appears that survival is somewhat improved in patients with UIP in RA-ILD as opposed to IPF, but 5-year survival is estimated to be less than 50% [28, 29]. In studies comparing HRCT findings with pathological confirmation of UIP, reticular markings and/or peripheral honeycombing are highly sensitive for UIP [24, 26]. There are a few instances in which HRCT features consistent with UIP were actually found to be fibrotic nonspecific interstitial pneumonitis (fibrotic NSIP) on pathological examination, but it is unclear if this distinction improves the prognosis [27, 30]. A study by Biederer et al. suggested that men and smokers were more likely to have UIP, with a trend toward purely reticular findings on HRCT as the number of pack years increases [31]. 

Pathologic findings consistent with NSIP were found to have predominantly ground glass infiltrates on HRCT. This accounts for about a third of the cases of RA-ILD [26]. NSIP is a pattern more often found in women and nonsmokers, and it suggests active alveolar inflammation. Consequently, there is usually a favorable response to immunosuppressive medications, and prognosis is much better with survival estimates greater than 80% at 5 years from diagnosis [32]. 

Bronchiolitis obliterans-organizing pneumonia (BOOP) occurs less frequently in about 10% of patients, and it carries a similarly good prognosis as NSIP [33]. Hallmark features on HRCT include subpleural consolidation with patchy ground glass infiltrates. All of the other ILD histologic subtypes have been reported in RA-ILD including lymphocytic interstitial lung disease (LIP), which may have peribronchiolar or centribular nodules and cystic lesions, as well as the more diffuse diseases including desquamative interstitial pneumonitis (DIP), acute interstitial pneumonitis (AIP), and respiratory bronchiolitis-interstitial lung disease (RB-ILD). 

Open lung biopsy is the only definitive procedure to accurately diagnose an interstitial lung disease, but this is rarely necessary in RA-ILD as suggested by the joint ATS-ERS statement regarding the evaluation of interstitial lung disease in collagen vascular diseases [34]. Additionally, there may be a 20% increase in mortality if an open lung biopsy is performed in the setting of an acute exacerbation of ILD [35]. We do occasionally employ transbronchial biopsies in conjunction with BAL in an attempt to exclude other coexisting infectious, granulomatous, or eosinophilic diseases, but we never make a determination of the histologic injury of an ILD based on this information. It is not uncommon to obtain a pathologic reading of NSIP although the leading edge of the injury or a biopsy from another lung segment may be more consistent with UIP [27]. In general, we focus on a thorough history to evaluate for other potential causes of the radiologic findings and then proceed with bronchoscopy for BAL with or without transbronchial biopsies to exclude an alternative diagnosis as described above.

## 5. Differential Diagnosis in Established Disease

Once a diagnosis has been rendered, the real challenge begins. As our index case for this paper illustrates, a clinical worsening in the context of established disease presents one of the most difficult aspects of care in RA-ILD. We generally define a clinical worsening as the onset of fever, cough, or new/progressive dyspnea on exertion, and/or a decline in the forced vital capacity (FVC) by 10% or the DLCO by 15% [34]. 

Infection remains the leading cause of death in RA, accounting for 20–30% of the mortality [36, 37]. Methotrexate is the most extensively reported medication associated with pulmonary drug toxicity, but almost every medication available for the treatment of RA has been implicated in the development of pneumonitis. An often, overlooked possibility for clinical worsening is environmental or occupational exposures. There are clinical characteristics that increase the likelihood of each of these possibilities in any given patient, and all of these need to be explored before determining that a clinical worsening is attributable to the underlying RA-ILD.

## 6. Infectious Complications

 RA patients on immunosuppressive therapy are at an increased risk of developing lower respiratory tract infections, particularly if they have pre-existing lung disease. Many infectious organisms, including *mycobacterium avium, mycobacterium tuberculosis*, and *pneumocystis jirovecii*, as well as fungal pathogens can have a radiologic appearance that mimics RA-ILD with increased ground glass and interstitial infiltrates. An additional consideration is that chronic prednisone use increases the risk of hospitalization for pneumonia in RA patients in a dose-dependent fashion with doses greater than 10 mg/day more than doubling the risk [38]. Another study highlighted an increased risk of pneumocystis pneumonia in patients taking as little as 15 mg of prednisone per day for more than 8 weeks suggesting that the use of trimethoprim-sulfamethoxazole as prophylaxis in these patients may be beneficial [39]. 

It is also important to note that reactivation of tuberculosis in the setting of anti-TNF-*α* agents can occur and manifest with pulmonary infiltrates, but it appears that extrapulmonary TB may be more common in this setting occurring in 65% of cases and manifesting as disseminated or hepatosplenic disease or even TB meningitis [40]. It is possible to develop disseminated tuberculosis on anti-TNF-*α* agents despite a negative PPD in patients already receiving mild immunosuppressive medications. This has prompted the British Thoracic Society to recommend an individual risk-benefit assessment in these patients based on the estimated annual risk of MTB multiplied by a factor of 5 [41]. If disseminated MTB is suspected, sampling multiple sites is recommended since the diagnostic yield from sputum or BAL culture is reported as 70% or less [42]. 

 Any suspicion of an infectious complication necessitates a prompt and thorough investigation. Fever is a common manifestation of drug-induced pneumonitis, and so it is not definitive evidence of an infectious complication. Immunosuppressive medications may prevent a robust leukocyte response to an infectious organism, so our approach to the patient with pulmonary infiltrates often incorporates early bronchoalveolar lavage in addition to routine blood and urine cultures. 

## 7. Drug-Induced Pneumonitis

A number of medications used to treat RA have been associated with the development of pulmonary toxicity. We will briefly describe the main pulmonary toxicities associated with each drug class and explore the risk factors for diffuse pneumonitis in the case of methotrexate and the anti-TNF-*α* biologic agents.  Table 2 is provided to summarize these toxicities.

**Environmental Toxins**

As our index case would suggest, a thorough review of potential environmental toxins needs to be explored. There are over 300 environmental exposures that can increase the odds of developing a hypersensitivity pneumonitis (extrinsic allergic alveolitis) [73]. Specific questions during the clinical history should address possible inhaled allergens in the home or work place including mold, dusts, chemicals, and pet dander. In particular, our patient kept cockatiels as house pets putting her at risk for bird fancier's lung. Other examples include farmer's lung caused by *thermophilic actinomycetes*, lab worker's lung due to rodent antigens, and epoxy-resin lung due to phtalic anhyhdride [73].

Less than 20% of people with known exposures actually develop extrinsic allergic alveolitis, but acute symptoms included dyspnea, cough, and malaise and fever in some patients [74]. Chronic exposure can lead to pulmonary fibrosis [74]. HRCT will often demonstrate bilateral ground glass opacities and/or centrilobular nodules similar to RA-ILD [75]. If there is a known exposure, blood tests for serum precipitins to the suspected antigen are indicated. The finding of poorly formed noncaseating granulomas in specimens obtained by transbronchial biopsy can be helpful since these are atypical in RA-ILD as well as MTX pneumonitis where BAL also shows a lymphocytosis similar to hypersensitivity pneumonitis [73]. If a diagnosis of hypersensitivity pneumonitis is rendered, antigen avoidance can be curative, but it often takes up to three weeks to recover from an acute episode, and chronic damage may be irreversible. Steroid therapy may hasten the improvement in symptoms.

**Malignancy**

The presence of RA-ILD increases the odds of developing lung cancer, but immunosuppression itself can also raise the cancer risk [28]. Diffuse infiltrates may actually represent hematogenous or more likely lymphangitic spread of an underlying malignancy. While this may be a rare complication, it is one that should be considered when a patient seemingly fails to respond to treatment. Additionally, BAL in combination with transbronchial biopsy may provide the correct diagnosis in cases of lymphangitic spread.

### 7.1. Nonsteroidal Anti-Inflammatory Drugs (NSAIDs)

In high doses, NSAIDs have been implicated in the development of diffuse pulmonary infiltrates. In approximately 10–15% of cases, these infiltrates were simply pulmonary edema, but often these infiltrates reflect an acute eosinophilic pneumonia [43]. In fact, a review by Goodwin and Glenny found naproxen to be the NSAID most commonly associated with acute eosinophilic pneumonia, but there were also cases associated with just any NSAID [44]. Thankfully, the acute inflammation associated with these medications does not typically translate into chronic fibrotic lung disease, and withdrawal of the offending agent with or without additional steroid therapy resolves the inflammatory response. 

### 7.2. Gold Salts and Penicillamine

Gold salts and penicillamine seem to have fallen out of favor with the advent of methotrexate and the newer biologic agents, but a few patients continue to use these medications. The classic pulmonary toxicities associated with these drugs include interstitial pneumonitis, bronchiolitis obliterans with or without organizing pneumonia, pulmonary renal syndromes, and diffuse alveolar damage [45–47].

### 7.3. Methotrexate

 The most frequently implicated RA drug leading to pulmonary toxicity is methotrexate (MTX), but the estimated risk of developing MTX pneumonitis may actually be relatively low at doses of 20–25 mg/week. One study found that only 3% of 130 patients followed for seven years actually developed MTX-induced pulmonary toxicity [48]. Unfortunately, the subset of patients developing MTX pneumonitis faced a possible 20% increased mortality rate especially if the drug toxicity occurred in the first six months of treatment [49]. Consequently, attempts to identify patients at risk for pneumonitis should be an important therapeutic goal. 

Saravanan and Kelly found that the greatest predictor for the development of pneumonitis is pre-existing pulmonary disease, with a baseline abnormality in the diffusing capacity less than 70% of predicted incurring a 10-fold increased risk of MTX drug toxicity [50]. Tobacco abuse, hypoalbuminemia, and previous use of DMARDs including sulfasalazine, gold salts, or D-penicillamine also have large attributable risks [51]. Minor risk factors including advanced age, diabetes mellitus, and RA pleuropulmonary involvement increase the risk for MTX pneumonitis [37, 52, 53]. Our current recommendation is to obtain pulmonary function tests prior to the institution of MTX, and if the DLCO is less than 70% of predicted, and an HRCT shows any evidence of pleural or interstitial thickening that another agent considered. 

If treatment has already commenced, several clinical parameters can be monitored for the development of acute MTX drug toxicity. Surprisingly, routine surveillance of PFTs is not useful in predicting drug toxicity, perhaps due to the acute onset of symptoms [54, 55]. However, duration of therapy has some clinical utility, since roughly half of patients that are going to develop MTX drug toxicity will be symptomatic within the first 6–8 months of treatment with 10% of those patients developing symptoms in the first 10 weeks [49]. Low-grade fever is the most prevalent symptom, reported in greater than 90% of patients. Roughly 80% of patients also developed a dry, nonproductive cough with the vast majority of these patients also reporting dyspnea on exertion [49]. 

Fever should prompt a thorough investigation for an infectious etiology, and if bronchoscopy is employed, a bronchoalveolar lavage cell count and flow cytometry can be useful. The lavage fluid will often show a shift away from a neutrophilic cell type to a predominantly lymphocytic infiltrate, with an increased CD4 : CD8 ratio [56, 57]. Additionally, a small study by Inokuma et al. revealed a decline in the serum absolute lymphocyte count below 500 cells/mm^3^ was closely associated with MTX drug toxicity [58]. Imaging with HRCT characteristically displays a diffuse interstitial pattern in greater than 93% of patients with MTX pneumonitis. Pleural thickening and less commonly pleural effusions were also found in a small subset of patients [49]. While it is exceedingly unnecessary, an open lung biopsy can help distinguish between RA-ILD and MTX pneumonitis. In general, MTX pneumonitis will have features of acute and organizing diffuse alveolar damage with cellular interstitial infiltrates with or without granulomas [59]. This pattern may also be seen in some infections but generally differs from the classic pathologic features of UIP and NSIP in RA-ILD. However, given the availability of other medications to treat RA, open lung biopsy is rarely necessary. 

Two other patterns of chronic injury have been reported as well. A few patients have developed an insidious alveolar fibrosis in the absence of systemic symptoms as well as a persistent chronic cough that were relieved with cessation of MTX therapy [57, 60]. Additionally, there are several studies that have shown a decrease in the FEV1 : FVC ratio and an increased residual volume as the most commonly reported findings on PFTs of patients on chronic MTX therapy [54, 61, 62]. 

If acute or chronic toxicity is suspected, withdrawal of MTX is indicated. Once infection has been excluded, treatment of the acute toxicity with supportive care is generally sufficient. In more severe or life-threatening cases, pulse dose glucocorticoids at 1 mg/kg/day can accelerate the resolution and should be strongly considered [63]. However, if the response to glucocorticoid therapy is inadequate, the addition of azathioprine or cyclophosphamide can dramatically improve the clinical response in some cases [64, 65]. If a patient develops acute toxicity, rechallenge with MTX is ill advised as the study by Kremer et al. uncovered mortality rates as high as 50% in this setting [49].

### 7.4. Leflunomide

 Leflunomide was initially reported to cause pneumonitis similar to MTX in Asian populations suggesting a genetic predisposition to pulmonary toxicity [66]. However, case reports from Australia, Germany, and the UK have expanded the awareness of pulmonary toxicity with leflunomide use [67]. In a comprehensive evaluation of the existing literature, pulmonary toxicity occurred within 12 weeks of the initiation of therapy in all patients that received a loading dose of leflunomide and most patients who had pre-existing ILD [67–69]. The mortality associated with the pneumonitis was reported as 19% predominantly occurring in the subset of patients with previously noted ground glass infiltrates on HRCT [67].

### 7.5. Anti-TNF-*α* Biologic Agents

There are case reports of patients initiated on treatment with infliximab, adalimumab, or etanercept who have developed rapidly progressive and sometimes fatal pulmonary fibrosis [70, 71]. Recently, data from the British Society for Rheumatology's Biological Register (BSRBR) revealed that while the overall mortality was no different between anti-TNF-*α* agents and DMARDs, mortality from ILD was nearly tripled in patients on anti-TNF-*α* therapy compared to control patients on DMARDs alone [72]. The authors listed some potential problems with the study that may account for that finding, but it appears that pulmonary toxicity associated with these medications is an increasingly recognized problem. More studies will be needed to determine which patients may be at risk for this complication.

## 8. Treatment of RA-ILD

Once a diagnosis of RA-ILD has been made, treatment is generally focused on controlling the systemic disease with immunosuppressive agents while tailoring therapy to the underlying histopathologic subtype. If characteristics of NSIP or BOOP predominate the radiologic appearance with ground glass infiltrates on HRCT, glucocorticoids alone may be effective [76]. We follow a standardized protocol set forth by Lazor et al. in which patients received 0.75 mg/kg/day prednisone during the initial four weeks of treatment, then 0.5 mg/kg/day for the next four weeks, then 20 mg/day for four weeks tapering to 10 mg/day for the next 6 weeks, and then 5 mg/day for 6 weeks [77].

A definitive response to treatment is defined as a 10% improvement in the FVC or 15% improvement in the DLCO at 12 weeks [32]. Based on recent data from Zappala et al. we continue therapy even if there is a marginal degree of improvement (5–10% in FVC or 10–15% in DLCO) [78]. If the patient meets criteria for improvement, we check thiopurine methyltransferase (TPMT) activity levels and if normal initiate steroid sparing therapy with azathioprine 50 mg daily gradually increasing the dose to 2-3 mg/kg/day [79]. If TPMT activity is decreased, cyclosporine is substituted at a dose of 2.5 mg/kg/day divided twice daily [80]. If the FVC and DLCO remain stable with quarterly PFT assessments over a two-year period, we consider this evidence that progression of disease has been halted, at which time it may be reasonable to reassess the medical regimen [32]. 

If there is evidence of reticular markings and honeycombing suggesting UIP, we still attempt a trial of immunosuppressive therapy as described above, but we also treat with N-acetylcysteine 600 mg three times daily based on clinical studies in idiopathic pulmonary fibrosis [81]. Cyclophosphamide (2 mg/kg/day orally) is initiated in conjunction with lower-dose prednisone (0.25 mg/kg/day) for steroid nonresponders [82]. If they respond, therapy is continued for 18 months to 2 years at which point the medical regimen may be reconsidered or therapy can continue indefinitely. Appropriately selected non-responders are referred for lung transplantation.

One additional challenge involves patients referred for an evaluation of ILD who have occult autoimmune disease. This affects about 10% of patients who ultimately go on to develop systemic features of rheumatoid arthritis [83]. If the anti-CCP antibodies are positive, we will treat the interstitial lung disease as delineated above and refer to rheumatology for further evaluation and management.

## 9. Treatment of an RA-ILD Exacerbation

An ILD exacerbation is generally defined as rapidly deteriorating respiratory symptoms within a 30-day period with evidence of new infiltrates (usually new ground glass opacities) and exclusion of an identifiable cause [84]. The absolute risk of an acute exacerbation is not well established, but based on the limited data of acute exacerbations in collagen vascular diseases, possibly as many as 20% of patients with RA-ILD will experience an acute exacerbation with a 1-year incidence as high as 2.58% [85]. By the time patients present to the hospital, hypoxemia may be severe and a terminal outcome may be eminent [85, 86].

Once alternative explanations for a clinical worsening, such as infection and/or drug reaction, have been excluded, treatment is dictated by the underlying histopathology. As common with many rare diseases, there is a paucity of well-designed clinical trials to guide therapy in RA-ILD, but if the underlying histopathology is NSIP or BOOP, there is a reasonable probability for a sustained clinical response to glucocorticoid treatment alone or in conjunction with other immunosuppressive agents [76]. However, ground glass infiltrates on the background of UIP often herald the development of diffuse alveolar damage, and the ensuing mortality approaches 80–100% [85–88]. The only evidence to support treatment in this cohort comes from a handful of isolated case reports, while more robust observational studies have demonstrated a consistently poor prognosis [85–88]. Given the lack of evidence to support immunosuppression in UIP, decisions to treat must be made on an individual basis.

We generally initiate treatment with IV methylprednisolone at a dose of 1 mg/kg/day for 3 days followed by oral prednisolone 1 mg/kg/day. If there is no evidence of improvement in paO_2_/FiO_2_ratio or oxygen saturation rescue therapy with cyclophosphamide (500–750 mg/m^2^) may be considered [89]. Other regimens have employed cyclosporine 3 mg/kg/day or tacrolimus 3 mg/kg/day in divided doses in patients unresponsive to cyclophosphamide with some success in isolated case reports [90, 91]. If the patient progresses to the point of requiring ventilatory support, consideration should be given to noninvasive ventilation, and/or lower PEEP settings and avoidance of recruitment maneuvers, since PEEP levels >10 cm H_2_O had more than a 4-fold associated risk of subsequent mortality in one study of acute ILD exacerbations [92]. 

## 10. Other Possible Drug Therapies

### 10.1. Anticoagulation

 In situ thrombosis within the pulmonary vasculature has been documented in ILD, and a trial of anticoagulation with warfarin by Kubo et al. demonstrated improved survival in this patient population [93]. This may be a reasonable medical therapy in patients with extensive RA-ILD although there are no specific trials investigating its use in this patient population.

### 10.2. N-Acetylcysteine (NAC)

 NAC is an antioxidant found to be mildly effective at preventing the decline in lung function in idiopathic pulmonary fibrosis (IPF) [81]. While there is no data regarding its use in RA-ILD, its side effect profile mirrors multivitamins. Since UIP is the predominant histopathology in RA-ILD and is the hallmark pathologic finding in IPF, a dose of 600 mg three times daily may be reasonable to consider in RA-ILD.

### 10.3. Mycophenolate

This medication has been used for the treatment of RA-ILD and it appears effective in patients with early or limited disease at a dose of 1-2 mg/day [94, 95]. It does not appear to be that effective in relieving articular manifestations of the disease, and therefore it needs to be used in conjunction with DMARD's in most cases. 

### 10.4. Rituximab

 This biologic agent is an anti-CD20 antibody licensed for the treatment of RA refractory to anti-TNF-*α* therapy. Interestingly, RA-ILD patients with a UIP histological pattern have increased numbers of CD-20 positive B cells aggregated around small airways [96]. Although there are no published trials on the use of rituximab for RA-ILD, Popa et al. found that RA patients treated with rituximab for 7 years have not shown any evidence of new RA-ILD [97]. There are also several cases reporting the successful use of weekly rituximab for ILD in other forms of collagen vascular disease [98–101].

The optimal treatment strategy remains elusive due to the rare nature of the disease. Hopefully, increased coordination among multiple academic centers will help advance the evaluation of novel medications in large, well-conducted, clinical trials.

## 11. Conclusion

It is becoming increasingly obvious that RA-ILD is a more prominent and debilitating manifestation of RA than once believed. As our index case demonstrated, there are host of factors that can obscure the diagnosis, but prompt diagnosis and treatment may delay the progression to end-stage lung disease. Unfortunately, once RA-ILD has been diagnosed, any acute worsening engages the clinician in an often, challenging evaluation process. Is it the disease, the medications, or some other unrelated but reversible factors contributing to a decline in lung function? 

In our patient, reticular markings and ground glass opacities were present in late 2007, but she did not experience significant symptoms for another several months. When she presented for evaluation, she already had a decline in her diffusing capacity to 61% of predicted. An infectious source was excluded, and it would have been very easy to attribute her symptoms to an obvious case of RA-ILD. However, given her environmental exposures, serum precipitins were drawn to evaluate for a hypersensitivity pneumonitis. She was found to have elevated IgG precipitins to cockatiels, and lymphocytosis was noted on bronchoalveolar lavage. Given a diagnosis of probable hypersensitivity pneumonitis she was started on high-dose prednisone, and she removed her cockatiels from the home. Over the next three months her DLCO improved to 89%. Her prednisone was tapered to 10 mg daily, and one year later her, DLCO had been normalized at 98% of predicted.

## Figures and Tables

**Table 1:** Respiratory complications of rheumatoid arthritis.

Lung structure	Disease manifestations
Lung parenchyma	Interstitial lung disease (ILD)
	Usual interstitial pneumonitis (UIP)
	Nonspecific interstitial pneumonitis (NSIP)
	Bronchiolitis obliterans with organizing pneumonia (BOOP)
	Lymphocytic interstitial pneumonitis (LIP)
	Desquamative interstitial pneumonitis (DIP)
	Diffuse alveolar damage (DAD)
	Drug-induced pneumonitis
	Rheumatoid nodules (necrobiotic nodules)
	Caplan's syndrome (silicosis associated with RA)
	Infectious complications

Airways	Chronic obstructive pulmonary disease (COPD)
	Bullous emphysema
	Bronchiectasis
	Obliterative bronchiolitis (Constrictive bronchiolitis)

Pleura	Pleuritis
	Pleural effusion
	Spontaneous pneumothorax

Vascular	Pulmonary hypertension
	Diffuse alveolar hemorrhage

Extrapulmonary	Diaphragm weakness
	Cricoarytenoid arthritis with extrathoracic obstruction

**Table 2:** Common pulmonary drug toxicities and associated risk factors associated with medications used to treat rheumatoid arthritis.

Medication	Pulmonary toxicity	Risk factors
NSAIDS	Noncardiogenic pulmonary edema,	High-dose treatment
	Acute eosinophilic pneumonia,	
	Interstitial pneumonitis	

Gold salts	Interstitial pneumoitis, bronchiolitis obliterans with or without organizing pneumonia, pulmonary renal syndrome, diffuse alveolar damage	Unknown, possible genetic association and cumulative ingestion >500 mg gold

D-penicillamine	Bronchiolitis obliterans, pulmona renal syndrome, diffuse alveolar damage	Unknown

Methotrexate	Acute interstitial pneumonitis, pulmonary fibrosis, pleural thickening, and chronic cough	DLCO < 70% of predicted; tobacco abuse >25 pack years; hypoalbuminemia; prior use of DMARDs; RA pleuropulmonary involvement; advanced age; diabetes mellitus

Anti-TNF-*α* biologic agents	Interstitial pneumoitis, rapidly progressive pulmonary fibrosis	Possible prior RA-ILD

Leflunomide	Interstitial pneumonitis	Loading dose of leflunomide; pre-existing ground glass infiltrates on HRCT
